# Unsupervised proteomic stratification of people living with HIV reveals inflammatory biotypes beyond conventional clinical classifications

**DOI:** 10.3389/fimmu.2026.1947773

**Published:** 2026-09-10

**Authors:** Norma Rallón, Clara Restrepo, Alejandra Manquillo, Carlos Pérez-Sánchez, Ignacio Mahillo, Sara Nistal, Aws Al-Hayani, Alfonso Cabello, Irene Carrillo, Laura Prieto, Miguel Górgolas, Juan C. López, Ana Muñoz, Vicente Estrada, José M. Benito

**Affiliations:** 1 HIV and Viral Hepatitis Research Laboratory, Instituto de Investigación Sanitaria Fundación Jiménez Díaz, Universidad Autónoma de Madrid (IIS-FJD, UAM), Madrid, Spain; 2 Hospital Universitario Rey Juan Carlos, Móstoles, Spain; 3 Cobiomic Bioscience, Córdoba, Spain; 4 Department of Statistics, Instituto de Investigación Sanitaria Fundación Jiménez Díaz, Universidad Autónoma de Madrid (IIS-FJD, UAM), Madrid, Spain; 5 Hospital Universitario Fundación Jiménez Díaz, Instituto de Investigación Sanitaria Fundación Jiménez Díaz, Universidad Autónoma de Madrid (IIS-FJD, UAM), Madrid, Spain; 6 Hospital General Universitario Gregorio Marañón, Madrid, Spain; 7 Hospital Clínico Universitario San Carlos, Madrid, Spain

**Keywords:** elite controllers, HIV control, immunology, inflammation, proteomics

## Abstract

**Background:**

Conventional HIV-1 management relies on clinical phenotypes, assuming a uniform inflammatory status and overlooking biological heterogeneity. We aimed to employ a high-throughput proteomic approach to stratify people living with HIV (PLWH) based on plasma inflammatory profiles and evaluate their association with comorbidity burden.

**Methods:**

This cross-sectional study included 30 PLWH (10 treatment-naïve, 10 ART-suppressed, and 10 elite controllers (EC)) and 10 seronegative controls. Unsupervised hierarchical clustering was applied to 87 inflammatory proteins (analyzed by proximity extension assay and ELISA). A Random Forest model stabilized with 1,000 trees identified the variables defining the biotypes. Comorbidity burden was assessed within the virally suppressed groups (ART-suppressed and EC).

**Results:**

Clustering revealed two distinct biological profiles independent of clinical classifications: a high-inflammation biotype (n=11) and a low-inflammation biotype (n=19). Within the treatment-naïve, elite controller, and ART-suppressed groups, 60%, 30%, and 20% of participants, respectively, were classified as belonging to the high-inflammation biotype. PDL1, CD40, TNF, LAP-TGFbeta1, and SLAMF1 were the top biological drivers of this separation. A trend for a higher comorbidity burden was observed in the high-inflammation biotype compared to the low-inflammation group (p=0.06).

**Conclusions:**

Plasma inflammatory profiling stratifies PLWH into distinct biological biotypes that bypass conventional classifications. The observed differences in comorbidity burden between inflammatory biotypes warrant further investigation in larger prospective studies to clarify their clinical relevance and potential implications for long-term health outcomes.

## Introduction

The widespread use of combined antiretroviral therapy (ART) has revolutionized the clinical course of HIV-1 infection, granting sustained viral suppression and extending life expectancy (1). Nevertheless, successful control of viral replication does not directly translate to complete immune restoration. A substantial proportion of patients still display biomarkers of persistent immune activation and systemic inflammation (2, 3).

This chronic inflammatory state represents a critical challenge in the contemporary management of people living with HIV (PLWH). Residual immune activation is independently associated with an increased risk of developing non-AIDS-defining comorbidities, including cardiovascular events, metabolic disorders, and neurocognitive impairment, even in patients under long-term virological suppression (2, 4, 5).

Elite controllers (EC) represent a natural model of functional control, being able to maintain undetectable viral loads in the absence of therapy (6). However, growing evidence indicates that many EC exhibit elevated levels of inflammatory biomarkers similar to or even higher than those observed in ART-treated individuals (7–11). These observations suggest that spontaneous virological control may not be sufficient to achieve true immune restoration, thereby raising questions about the future development of non-AIDS events in this subset of patients.

To date, the vast majority of studies evaluating systemic inflammation and comorbidity burden in PLWH have categorized participants based on their clinical phenotypes (treatment-naïve, ART-suppressed, or elite controllers) (7–16). This rigid classification assumes that individuals within the same clinical group share a uniform inflammatory status, potentially overlooking the biological heterogeneity that exists regardless of therapeutic or spontaneous viral control.

To overcome this limitation, we designed a study encompassing a balanced proportion of the three distinct clinical phenotypes of the infection: active replication, pharmacological control, and spontaneous control. The originality of our approach lies in shifting the paradigm: rather than segregating the cohort by conventional clinical categories, we stratified the entire population according to their plasma inflammatory profiles using high-throughput proteomics, under the hypothesis that the risk of comorbidities is driven by the specific biological inflammatory biotype of the patient. Therefore, the objective of this study was to employ a high-throughput proteomic approach to stratify PLWH according to their plasma inflammatory profiles and evaluate its association with comorbidity burden.

## Methods

### Study design and participants

This cross-sectional study included adult participants with chronic HIV infection (PLWH) and HIV-seronegative individuals as uninfected controls (UC). The clinical characteristics and recruitment of this strictly balanced cohort have been previously described elsewhere (17); however, the current study applies an unsupervised hierarchical clustering to stratify patients by continuous proteomic variables rather than conventional clinical labels. To evaluate a broad spectrum of clinical presentations, the PLWH cohort was established with a balanced proportion of three distinct clinical phenotypes: a) active replication: treatment-naïve individuals with detectable plasma viral load (pVL) (offART group); b) ART-mediated control: non-controllers on ART with sustained undetectable pVL for at least five years (onART group); c) spontaneous control: elite controllers (EC) maintaining undetectable pVL in the absence of therapy (EC group).

Participants in the EC, onART, and UC groups were matched for age and sex. However, matching was not feasible for the offART group, which consisted entirely of male participants. This imbalance reflects current epidemiological trends of new HIV diagnoses in Spain, predominantly occurring within the men who have sex with men (MSM) population, as no treatment-naïve women were identified during the 12-month recruitment period (June 2022 to June 2023). Participants in the offART group were enrolled at the time of HIV diagnosis and prior to initiation of antiretroviral therapy, in accordance with current treatment recommendations advocating immediate ART initiation after diagnosis. Data allowing formal classification as acute or primary HIV infection were not available. All participants provided written informed consent. The study protocol was approved by the Ethical Review Board of the Instituto de Investigación Sanitaria-Fundación Jiménez Díaz (Madrid, Spain; Approval ID: PIC014-20_FJD) and conducted in accordance with the Declaration of Helsinki.

### Plasma samples

Venous blood samples were obtained via venipuncture in EDTA tubes and gently inverted to ensure adequate anticoagulation. Plasma separation was achieved by centrifugation at 2,000 × g for 10 minutes at room temperature. The resulting plasma was aliquoted into polypropylene tubes and stored at −20 °C until the proteomic and ELISA assays were performed. Plasma aliquots were stored at −20 °C for less than one year prior to proteomic analysis. All samples were thawed only once at the time of biomarker determination, and no repeated freeze-thaw cycles occurred before analysis. Samples from all study groups were collected, processed, and stored following identical procedures.

### High-throughput proteomic profiling and ELISA

Plasma inflammatory proteins were quantified using a high-sensitivity Proximity Extension Assay (PEA). This dual-recognition immunoassay utilizes antibody pairs linked to unique DNA oligonucleotides that hybridize upon binding to the target protein, generating a double-stranded DNA barcode quantified by qPCR. Olink technology incorporates an internal quality-control system designed to monitor both assay and sample performance and includes four internal controls added to each sample. These controls assess the three critical steps of the assay workflow (immunoreaction, extension, and amplification/detection), while normalization procedures are applied to reduce systematic technical variation and sample-processing noise. This method simultaneously assessed the relative expression of 92 distinct inflammatory markers. To maintain data quality, proteins with expression levels below the limit of detection across more than half of the samples, as well as sample outliers, were excluded prior to analysis.

An additional panel of nine proteins with established relevance to chronic inflammation, immune activation, microbial translocation, endothelial dysfunction, and coagulation in HIV infection, but not included in the Olink^®^ Target 96 Inflammation panel due to technical issues, was evaluated using commercially available enzyme-linked immunosorbent assays (ELISA). This targeted panel included sTNFRI, sTNFRII, hsCRP, sCD14, sICAM1, sVCAM1, FABP2, PAI1, and D-dimer. All assays were conducted strictly following the manufacturers’ instructions.

### Data stratification and statistical analysis

Baseline clinical and epidemiological characteristics across the balanced groups were expressed as median and interquartile range (IQR), with differences tested using non-parametric Kruskal-Wallis tests. The normalized protein expression (NPX) values derived from the PEA platform were log2-transformed for downstream interpretation.

To shift the focus from conventional clinical categories to biological profiles, unsupervised hierarchical clustering was applied to the standardized protein expression levels (Z-scores). Based on the resulting dendrograms, participants were classified into low and high inflammation biotypes. Principal Component Analysis (PCA) was subsequently used to visualize the clustering structure and explore the discriminatory capabilities of the protein expression between both groups.

Differential protein expression analyses were subsequently performed to identify proteins associated with the identified inflammatory biotypes. Protein expression levels were compared between the low- and high-inflammation groups using the non-parametric Wilcoxon rank-sum test. To account for multiple comparisons across the 87 proteins analyzed, p-values were adjusted using the Benjamini-Hochberg false discovery rate (FDR) procedure. Proteins with an FDR-adjusted p-value < 0.05 were considered significantly differentially expressed.

To identify the proteins contributing most strongly to the separation between the inflammatory biotypes, feature selection was performed using the Boruta algorithm followed by a Random Forest analysis stabilized with 1,000 trees. Variable-importance measures derived from the Random Forest model were used to rank the proteins according to their contribution to cluster separation. Finally, for the targeted ELISA data, generalized linear models (GLMs) were fitted to estimate adjusted mean levels across the defined biotypes, incorporating sex and age as covariates to minimize potential confounding effects.

## Results

### Characteristics of the study cohort

A total of 40 participants were included in the study, comprising 10 HIV-seronegative uninfected controls (UC) and 30 people living with HIV (PLWH). To ensure a balanced and representative evaluation of the entire disease spectrum, the PLWH cohort was established with a strictly proportional 1:1:1 design consisting of 10 offART participants with active replication, 10 onART participants with sustained viral suppression, and 10 elite controllers (EC) maintaining undetectable plasma viral loads (pVL) in the absence of therapy. This balanced design was strictly maintained despite the exceptional rarity of the EC phenotype in our clinical setting. Baseline characteristics across all groups are detailed in **Table 1**. While all study groups were similar in age, differences were observed in sex distribution. The UC, onART, and EC groups displayed comparable sex distributions, whereas the offART group consisted entirely of male participants. This specific imbalance directly reflects current epidemiological trends of new HIV diagnoses in Spain, which predominantly occur within the men who have sex with men (MSM) population, as no treatment-naïve women were identified during the 12-month recruitment period (June 2022 to June 2023).

**Table 1:** Characteristics of groups included in the study.

Characteristic	EC (n=10)	On ART (n=10)	off ART (n=10)	UC (n=10)	p-value
Age (years)	56 [45 – 61]	55 [47 – 60]	41 [34 – 52]	51 [45 – 57]	0.120
Sex (% of males)	60	60	100	60	0.126
Years since HIV diagnosis	19 [11– 24]	13 [8 – 20]	0	NA	**<0.0001**
Years as EC	17 [6 – 23]	NA	NA	NA	NA
Years on ART	NA	7 [6 – 9]	NA	NA	NA
Plasma HIV load (copies/mL)	20	20	60900 [6073 - 568000]	NA	NA
CD4 count (cells/μL)	869 [514 – 1200]	993 [655 – 1121]	621 [335 – 744]	NA	**0.020**
Nadir CD4 count (cells/ μL)	703 [426 – 849]	533 [261 – 711]	621 [335 – 744]	NA	0.265

Data are given as median [Q1 – Q3], except sex that is expressed as percentage. Statistical differences between the four study groups were tested by Kruskall-Wallis test for continuous variables and by Chi-square test for sex. Statistical significant was considered for p-values below 0.05 (in bold in the table). NA, not apply.

### Identification of distinct inflammatory biotypes in PLWH

**Figure 1:**
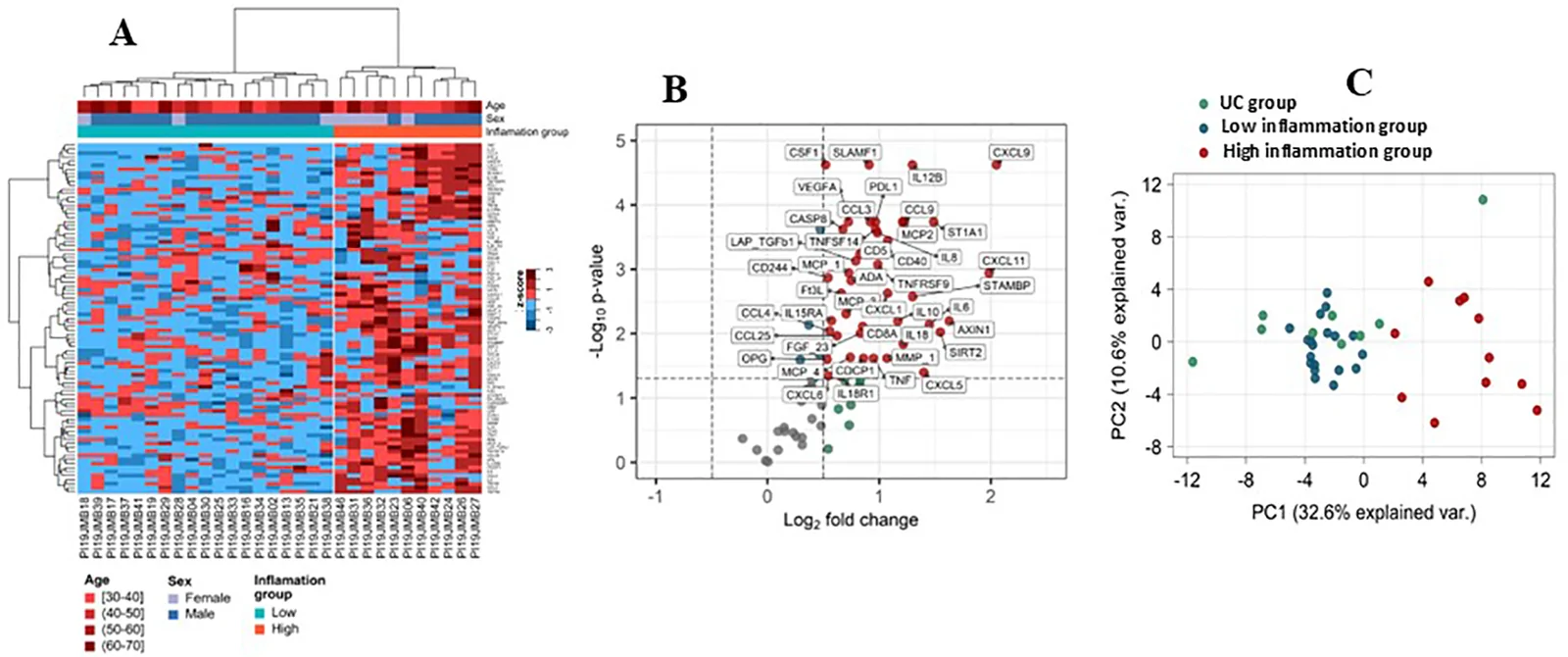
**(A)** Hierarchical clustering of PLWH (people living with HIV) participants (n=30) using the concentration (NPX-normalized protein expression) of 78 different plasma inflammatory proteins assayed by PEA (proximity extension assay), along with 9 proteins assayed by ELISA (enzyme linked immunosorbent assay). Color code in the heatmap indicates the Z-score of the concentration value for each protein, with red and blue colors indicating high and low relative concentration respectively. Age, sex and the inflammation group are color coded and its distribution across the cohort is shown at the top of the heatmap. In the heatmap, each file represents a protein and each column a PLWH participant (participant code is shown at the bottom of the heatmap). **(B)** Volcano plot showing the differential expression (DE) of inflammatory proteins between the low- and high-inflammation groups of PLWH participants. Differential expression was assessed using the non-parametric Wilcoxon rank-sum test, and p-values were adjusted for multiple comparisons using the Benjamini-Hochberg false discovery rate (FDR) procedure. Red dots indicate proteins significantly increased in the high-inflammation group compared with the low-inflammation group (FDR-adjusted p-value < 0.05). **(C)** Principal component analysis (PCA) of plasma inflammatory proteins using the first two principal components. Distribution of PCA values for PLWH participants of the high and low inflammation groups as well as for participants of the uninfected (UC) group are shown.

**Figure 2:**
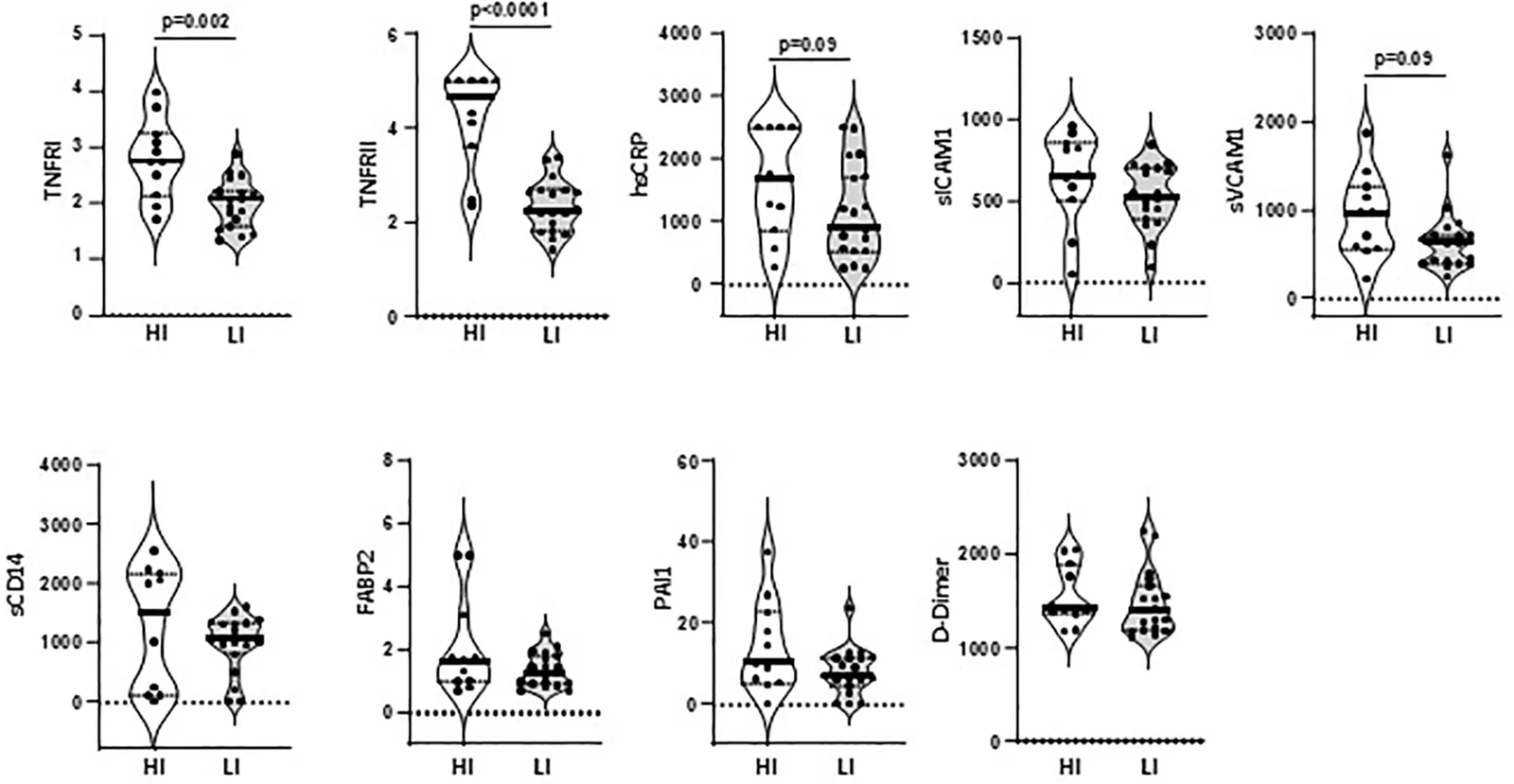
Violin-plots displaying the levels of different inflammatory markers assayed by ELISA (enzyme linked immunosorbent assay) in two groups of PLWH (people living with HIV) according to the degree of inflammation: high inflammation (HI) and low inflammation (LI) groups. Y-axis shows the concentration of each marker in ng/mL. Mann-Whitney U test was used for the pairwise comparisons. Only p-values lower than 0.1 are shown.

To explore the biological heterogeneity beyond conventional clinical classifications, unsupervised hierarchical clustering was applied to the relative expression levels of the detected plasma proteins. Of the 92 proteins assessed by the proximity extension assay, 14 were excluded because more than half of the samples were below the limit of detection. Consequently, the clustering process was executed using the remaining 78 Olink proteins alongside the 9 inflammatory proteins evaluated by ELISA. This analysis revealed the presence of two distinct biological clusters among PLWH: a high-inflammation biotype consisting of 11 participants and a low-inflammation biotype comprising 19 participants (**Figure 1A**). Differential expression analyses identified 51 proteins that remained significantly associated with the inflammatory biotypes after correction for multiple testing using the Benjamini-Hochberg false discovery rate (FDR) procedure (adjusted p-value < 0.05). (**Figure 1B**; **Supplementary Table 1**). Principal Component Analysis (PCA) confirmed a clear spatial separation between both biotypes, with the low-inflammation group clustering closely with the HIV-seronegative uninfected controls (**Figure 1C**). Interestingly, the high-inflammation biotype was not exclusively composed of patients with active viral replication; while it encompassed 60% of the offART group (6/10), it also included 30% of the EC (3/10) and 20% of the onART (2/10) participants. Participants assigned to the high-inflammation biotype exhibited elevated adjusted mean levels of TNFRI, TNFRII, hsCRP, and sVCAM1 compared to the low-inflammation group (**Figure 2**). To assess the potential influence of sex imbalance in the offART group on cluster formation, we performed an additional unsupervised clustering analysis restricted to the virally suppressed participants (EC and onART groups), which were matched for age and sex. This analysis again identified two distinct inflammatory biotypes and reproduced the classification of all five participants assigned to the high-inflammation biotype in the original analysis (three EC and two onART participants), supporting the robustness of the clustering results independently of the offART subgroup (**Supplementary Figure 1**).

**Figure 3:**
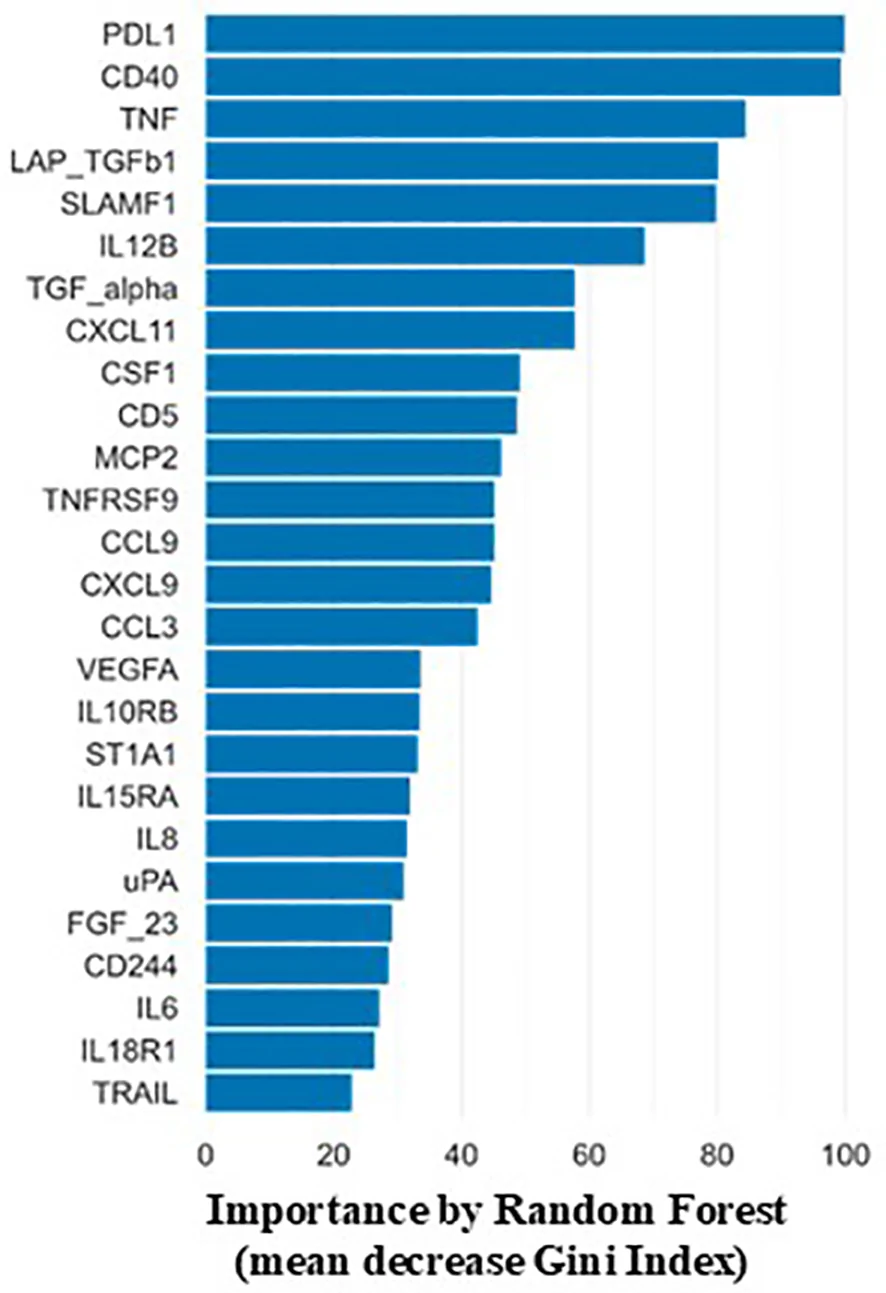
Identification of the inflammatory proteins contributing most strongly to separation between the high- and low-inflammation biotypes. Bars represent the relative importance of each protein, as estimated by the mean decrease in the Gini Index in the Random Forest variable-importance analysis performed after Boruta feature selection. Proteins with higher scores contributed more strongly to the discrimination between the two inflammatory biotypes.

To ascertain the specific proteins driving the discrimination between both biotypes, feature selection was performed using the Boruta algorithm followed by a Random Forest variable-importance analysis. PDL1, CD40, TNF, LAP-TGFβ1, and SLAMF1 emerged as the top five proteins contributing most strongly to the separation between the high- and low-inflammation biotypes (**Figure 3**). Interestingly the Random Forest variable-importance analysis restricted to the virally suppressed participants (EC and onART groups), yielded the same results with CD40, PDL1, IL6, LAP-TGFβ1, and TNF emerging as the principal contributors to the separation between both biotypes (**Supplementary Figure 1**).

### Inflammatory biotypes and comorbidity burden

**Figure 4:**
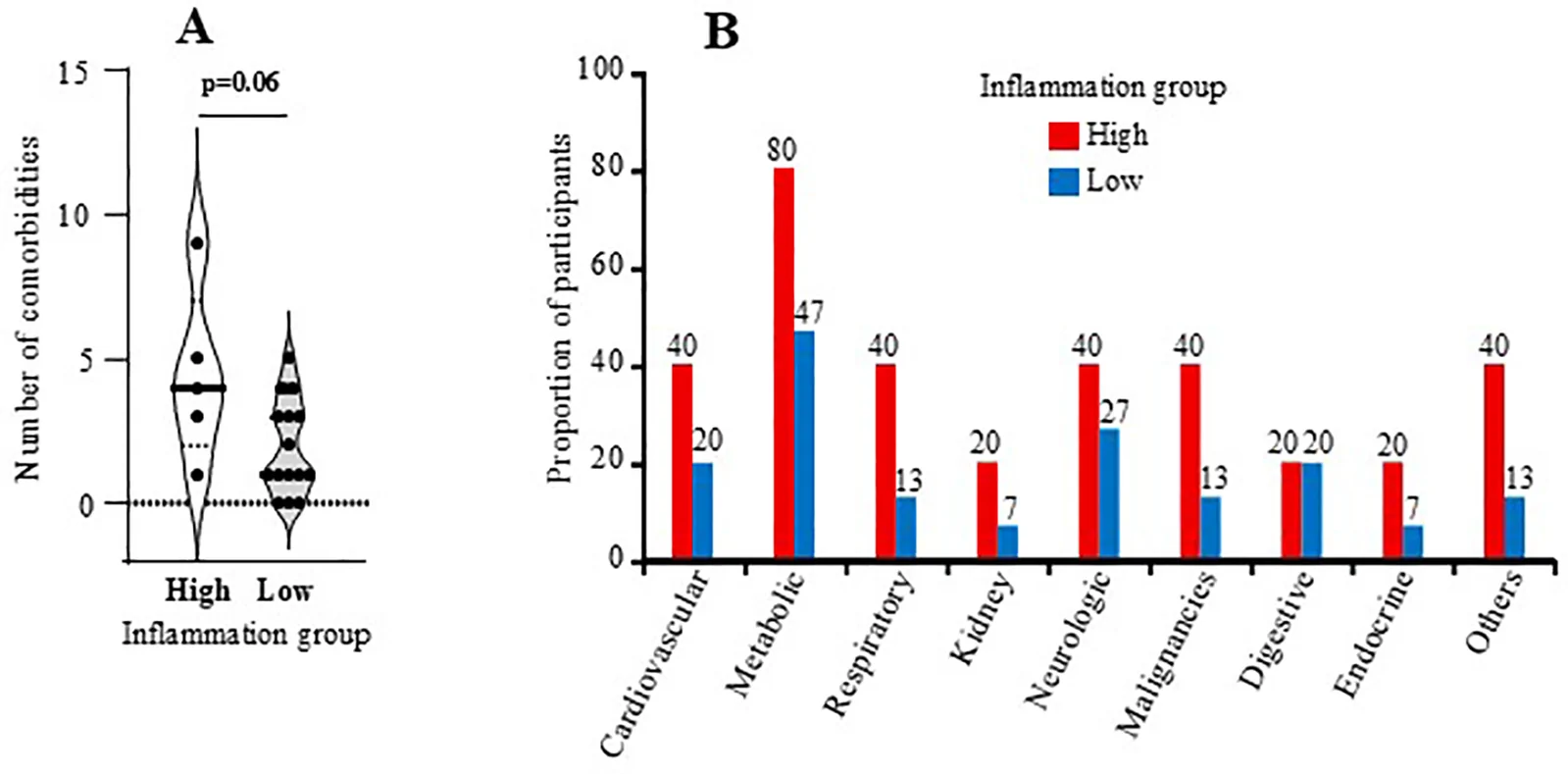
**(A)** Violin-plots displaying the burden of comorbidities (represented as number of comorbidities) among PLWH with undetectable levels of HIV viremia (EC and onART groups) at the time of inclusion in the study, according to degree of inflammation (high versus low). Mann-Whitney U test was used for the pairwise comparisons. **(B)** Bar graph showing the frequency (proportion of participants) of different comorbidities in PLWH (people living with HIV) with undetectable levels of HIV viremia (EC and onART groups) according to the degree of inflammation (high versus low). The number on the top of each bar indicates the proportion of participants showing each type of comorbidity.

At the time of study enrollment, we documented the presence of a wide spectrum of clinical comorbidities categorized across cardiovascular, metabolic, respiratory, renal, neurological, neoplastic, digestive, and endocrine systems. To assess the clinical impact of the inflammatory profile, the comorbidity burden, quantified as the total number of distinct clinical conditions per participant, was specifically evaluated within the onART and EC groups. The offART group was excluded from this specific analysis since their time since HIV diagnosis was zero at the moment of enrollment. The overall median (IQR) number of comorbidities in this population was 2.5 (1–4). When participants were stratified by their biological profile rather than their clinical phenotype, a trend for a higher comorbidity burden was observed in the high-inflammation biotype compared to the low-inflammation group (p=0.06; **Figure 4A**). Furthermore, a greater proportion of participants within the high-inflammation biotype exhibited distinct cardiovascular, metabolic, and neurocognitive conditions (**Figure 4B**), although statistical significance was limited by the small size of the sub-cohorts (5 PLWH in the high-inflammation group and 15 in the low-inflammation group).

## Discussion

The clinical management of HIV-1 infection has historically relied on rigid phenotypic classifications based on treatment status and viral suppression. However, the findings of the present study challenge this conventional paradigm by demonstrating that clinical labels fail to reflect the true underlying biological heterogeneity of people living with HIV. By shifting the focus from therapeutic categories to unsupervised hierarchical clustering of 87 distinct inflammatory proteins, we identified two natural, well-defined plasma inflammatory biotypes that cut across traditional clinical boundaries, in agreement with a previous study performed in a large population of PLWH on ART (18). Strikingly, a substantial proportion of individuals with sustained virological control—including both pharmacologically suppressed patients and elite controllers—co-segregated within the high-inflammation biotype alongside treatment-naïve participants exhibiting active viral replication (4, 19). This observation strongly suggests that spontaneous or therapy-induced suppression of plasma viremia does not automatically translate into complete immune reconstitution (19–21), revealing a persistent, silent inflammatory state that may remain masked under the veil of undetectable viral loads.

To further dissect this heterogeneity, the application of feature selection through the Boruta algorithm and a Random Forest variable-importance analysis allowed us to ascertain the specific proteins driving the discrimination between both biotypes. Among the 87 analyzed proteins, PDL1, CD40, TNF, LAP-TGFβ1, and SLAMF1 emerged as the top five biological contributors driving the separation between the high and low inflammation profiles. The proteins driving the separation between inflammatory biotypes are consistent with biological pathways previously implicated in chronic immune activation during HIV infection. TNF is a central mediator of systemic inflammation and has been repeatedly associated with immune activation, tissue damage, and the development of non-AIDS comorbidities in PLWH (22). Likewise, CD40 signaling plays a key role in antigen-presenting cell activation and amplification of inflammatory responses, contributing to persistent immune dysregulation in HIV infection (23). PDL1 reflects the chronic antigenic stimulation characteristic of HIV infection and has been linked to T-cell exhaustion and impaired immune function (24). Other immunoregulatory mediators not included in our panel, such as Galectin-9, have also been associated with chronic inflammatory states and tumor-promoting immune regulation (25). In contrast, LAP-TGFβ1 is involved in the regulation of immunosuppressive and tissue remodeling pathways (26), while SLAMF1 participates in the modulation of both innate and adaptive immune responses (27). Together, these findings support the concept that the identified inflammatory biotypes are characterized by a complex interplay between immune activation, immune regulation, and chronic inflammatory signaling, and align with the known biology of chronic HIV infection, where immune checkpoint molecules and tumor necrosis factor pathways remain notoriously perturbed despite virological control (28, 29). The identification of these specific inflammatory pathways provides a strong rationale for the future development of targeted therapeutic strategies aimed at attenuating systemic inflammation, such as agents targeting immune checkpoints, cytokine signaling, or microbial translocation (30).

The present study focused on circulating inflammatory proteins as minimally invasive blood-based biomarkers capable of identifying biologically distinct inflammatory profiles among PLWH. Beyond proteomic profiling, emerging liquid biopsy and integrative multi-omics approaches are increasingly being explored to improve disease stratification and biomarker discovery (31). In particular, circulating cell-free DNA and epigenetic signatures, including cfDNA methylation profiles, may provide complementary biological information and help capture disease heterogeneity that cannot be fully explained by a single molecular layer (32). Likewise, advances in proteomic technologies have facilitated the identification of novel immune pathways and potential therapeutic targets, supporting the concept that integrated molecular characterization may improve our understanding of complex immune-mediated conditions (33). Future studies combining proteomic, genomic, and epigenetic biomarkers could therefore contribute to a more comprehensive characterization of persistent inflammation and immune dysregulation in PLWH and help refine precision medicine strategies.

Furthermore, we observed a trend toward a higher burden of comorbidities among PLWH assigned to the high-inflammation biotype. This association, although limited by sample size, is consistent with previous evidence linking chronic inflammation to the development of non-AIDS comorbidities, including cardiovascular, metabolic, and neurocognitive disorders (3, 34, 35). In fact, recent longitudinal data from a Spanish cohort have demonstrated that specific panels of inflammatory and coagulation biomarkers are not only associated with, but also predictive of, non-AIDS-defining events in virologically suppressed PLWH (36). A critical consideration in our analysis is the potential for a bidirectional relationship between systemic inflammation and non-AIDS comorbidities. In the subgroup of PLWH with a high comorbidity burden, the elevated levels of pro-inflammatory molecules may not be exclusively driven by HIV-related factors. Clinical conditions such as obesity, renal impairment, and previous neoplasms are well-established independent drivers of chronic inflammation (37, 38). Therefore, the proteomic signatures identified in these individuals likely reflect a cumulative inflammatory state where HIV-specific immune activation and the physiological impact of co-existing pathologies synergize.

Furthermore, comorbidity burden was assessed as the total number of documented clinical conditions rather than using validated composite comorbidity indices. Given the exploratory nature of the study, the limited sample size of the subgroup analyzed, and the broad spectrum of age-related and HIV-associated conditions observed in the cohort, we considered this approach more appropriate for capturing overall multimorbidity. Although established instruments such as the Charlson Comorbidity Index or the Veterans Aging Cohort Study (VACS) Index may provide prognostic information, several conditions potentially relevant to chronic inflammation in people living with HIV are not fully represented in these scoring systems. Future studies in larger cohorts could evaluate whether the inflammatory biotypes identified here are also associated with validated prognostic comorbidity indices.

The distinct inflammatory signature identified in a subset of elite controllers carries important clinical implications. While elite controllers have traditionally been spared from treatment due to their ability to maintain undetectable viral loads spontaneously, our data showing that 30% of them exhibit a high-inflammation biotype supports accumulating evidence that persistent inflammation may contribute to an increased risk of non-AIDS comorbidities (8–10, 39). This aligns with previous reports showing that EC often display heightened immune activation and inflammation (8–10), possibly as a result of persistent antigenic stimulation or immune surveillance mechanisms (14–16). Taken together, these findings highlight the need for larger prospective studies to determine whether inflammatory biotypes have clinical relevance and whether inflammatory profiling may contribute to the identification of PLWH with different risks of long-term comorbidity burden.

Despite the novel insights provided, the present study has certain limitations that warrant caution when interpreting the results. First, the sample size is relatively small, which may limit the generalizability of the findings and the statistical power to detect smaller differences between groups, such as the trend toward a higher burden of comorbidities among individuals with elevated inflammation. However, the inclusion of the rare Elite Controller phenotype and the use of high-sensitivity proteomic profiling offer valuable exploratory insights into these distinct groups. Second, the cross-sectional design precludes any conclusions regarding the temporal stability of the identified inflammatory signatures or the establishment of causal relationships. Third, while our clustering approach revealed biologically meaningful subgroups, external validation in larger and more diverse cohorts is necessary to confirm these patterns. Fourth, the inflammatory biotypes and associated protein signature were identified and explored within the same dataset. Therefore, independent external validation cohorts will be required to confirm the robustness and generalizability of the identified inflammatory profiles. Fifth, neither the identification of inflammatory biotypes nor the exploratory analysis of comorbidity burden accounted for several potentially relevant confounding factors, including smoking status, obesity, HIV disease duration, CD4-related parameters, antiretroviral treatment characteristics, chronic viral coinfections, and other demographic or clinical variables. Consequently, both the observed clustering patterns and the trend toward a higher comorbidity burden in the high-inflammation biotype should be interpreted with caution, until confirmed in larger cohorts with adequate multivariable adjustment. Sixth, sex-related differences in inflammatory proteomic profiles may have influenced the observed clustering patterns. Although the clustering analysis performed in age- and sex-matched virally suppressed participants reproduced the main findings in the full cohort, residual confounding by sex cannot be fully excluded and should be addressed in larger validation cohorts. Finally, offART participants were enrolled at the time of HIV diagnosis, whereas elite controllers and onART individuals represented long-term stages of infection. Although the main findings were reproduced in the clustering analysis restricted to virally suppressed participants, the potential influence of disease stage and infection duration on the identified inflammatory biotypes cannot be completely excluded.

In summary, our study reveals that plasma inflammatory profiling can stratify people living with HIV into distinct biological biotypes that operate independently of conventional clinical classifications. The identification of specific proteins such as PDL1, CD40, and TNF as major drivers of this stratification, along with the observed trend toward a higher comorbidity burden in the high-inflammation group, underscores the potential of proteomic approaches for improving our understanding of biological heterogeneity in HIV. The observed differences in comorbidity burden between inflammatory biotypes warrant further investigation in larger prospective studies to clarify their clinical relevance and potential implications for long-term health outcomes.

## Data Availability

The original contributions presented in the study are included in the article/**Supplementary Material**. Further inquiries can be directed to the corresponding author/s.
