# Supplementary material for: Unsupervised proteomic stratification of people living with HIV reveals inflammatory biotypes beyond conventional clinical classifications
Source: Front Immunol. 2026 Sep 10;17:1947773. doi: 10.3389/fimmu.2026.1947773 (PMC13602527; doi:10.3389/fimmu.2026.1947773)
Supplement: Supplementary file 1 [file Table1.docx]

**Supplementary Table 1.** Differential expression analysis of inflammatory proteins in PLWH stratified according to inflammation biotype (high-inflammation versus low-inflammation groups). Results are presented as log2 fold-change (high inflammation versus low inflammation) and Benjamini-Hochberg false discovery rate (FDR)-adjusted p-values derived from Wilcoxon rank-sum tests.

| **Protein** | **Log2FC** | **P-value** |
| --- | --- | --- |
| SLAMF1 | 0.909 | 0.000 |
| IL12B | 1.298 | 0.000 |
| CSF1 | 0.520 | 0.000 |
| CXCL9 | 2.050 | 0.000 |
| MCP2 | 1.208 | 0.000 |
| VEGFA | 0.724 | 0.000 |
| CCL9 | 1.226 | 0.000 |
| CCL3 | 0.921 | 0.000 |
| ST1A1 | 1.489 | 0.000 |
| PDL1 | 0.967 | 0.000 |
| TNFSF14 | 0.966 | 0.000 |
| TGF_alpha | 0.473 | 0.000 |
| CASP8 | 0.675 | 0.000 |
| IL8 | 0.984 | 0.000 |
| CD40 | 1.076 | 0.000 |
| IL10RB | 0.476 | 0.000 |
| uPA | 0.441 | 0.000 |
| CD5 | 0.827 | 0.001 |
| LAP_TGFb1 | 0.790 | 0.001 |
| TNFRSF9 | 0.988 | 0.001 |
| CXCL11 | 1.984 | 0.001 |
| MCP_1 | 0.728 | 0.001 |
| CD244 | 0.542 | 0.001 |
| ADA | 0.749 | 0.002 |
| Ft3L | 0.660 | 0.002 |
| CXCL1 | 1.076 | 0.002 |
| STAMBP | 1.300 | 0.003 |
| MCP_3 | 0.704 | 0.005 |
| IL15RA | 0.573 | 0.006 |
| IL10 | 1.167 | 0.006 |
| AXIN1 | 1.627 | 0.006 |
| IL6 | 1.449 | 0.007 |
| CX3CL1 | 0.370 | 0.007 |
| CD8A | 0.852 | 0.008 |
| CCL4 | 0.555 | 0.009 |
| SIRT2 | 1.547 | 0.009 |
| FGF_23 | 0.832 | 0.010 |
| CCL25 | 0.623 | 0.011 |
| IL18 | 1.216 | 0.015 |
| CCL23 | 0.465 | 0.021 |
| MCP_4 | 0.741 | 0.023 |
| TNF | 0.949 | 0.024 |
| MMP_1 | 1.070 | 0.024 |
| CDCP1 | 0.860 | 0.024 |
| OPG | 0.534 | 0.025 |
| LIF_R | 0.294 | 0.026 |
| CXCL5 | 1.401 | 0.040 |
| TNFB | 0.363 | 0.041 |
| IL18R1 | 0.646 | 0.045 |
| CXCL6 | 0.547 | 0.045 |
| HGF | 0.430 | 0.047 |
